# Improved Models of Coalescence Ages of Y-DNA Haplogroups

**DOI:** 10.3390/genes12060862

**Published:** 2021-06-04

**Authors:** Iain McDonald

**Affiliations:** 1 Centre for Lifelong Learning, University of Strathclyde, 40 George St., Glasgow G1 1QE, UK; iain.mcdonald-2@manchester.ac.uk or iain.mcdonald@open.ac.uk; 2 Jodrell Bank Centre for Astrophysics, University of Manchester, Manchester M13 9PL, UK; 3 Department of Physics & Astronomy, Open University, Walton Hall, Milton Keynes MK7 6AA, UK

**Keywords:** human Y chromosome, molecular clock, male history, time estimation

## Abstract

Databases of commercial DNA-testing companies now contain more customers with sequenced DNA than any completed academic study, leading to growing interest from academic and forensic entities. An important result for both these entities and the test takers themselves is how closely two individuals are related in time, as calculated through one or more molecular clocks. For Y-DNA, existing interpretations of these clocks are insufficiently accurate to usefully measure relatedness in historic times. In this article, I update the methods used to calculate coalescence ages (times to most-recent common ancestor, or TMRCAs) using a new, probabilistic statistical model that includes Y-SNP, Y-STR and ancilliary historical data, and provide examples of its use.

## 1. Introduction

The human Y-DNA phylogenic tree is a uniquely written history of human relatedness and migration. Unlike autosomal DNA, which mixes within a population, Y-DNA unambiguously traces a family’s male line. Tests are commercially sold to trace the social and migratory history of patrilineal surnames. This can help connect individuals studying shared surname history, identify a misattributed parent or a birth father, or provide an identity to human remains. On a wider scale, multiple families can be linked to a common ancestor, tracing the migratory history of entire cultures and tying early historical genealogies to living families. A key feature of the male-specific portion of the Y-chromosome (MSY) is the accumulation of mutations throughout time, hence the number of mutations separating individuals can be translated into a time to most-recent common ancestor (TMRCA) via a molecular “clock”. Haplogroups, defined by related genetic testers within the Y-DNA tree, can similarly be defined by their coalescence age, representing the time into the past at which the common ancestor of those testers lived. In sufficiently widely tested haplogroups, this provides the haplogroup’s progenitor, the most-recent ancestor from whom all living men in that haplogroup are descended. In archaeology, such exploration of Y-DNA relationships can be used to reconstruct relatedness and migration during periods where ancient DNA cannot be sampled, e.g., because of the practice of cremation.

The two principal MSY molecular clocks are single-nucleotide polymorphisms (SNPs) and short tandem repeats (STRs). Literature TMRCA estimates initially focussed on Y-STRs, due to their low cost and availability of mutation rates. Bayesian TMRCA mutation models include the ‘infinite alleles method’, which calculates the probability of receiving the observed number of mis-matching Y-STR alleles in a given time, and the ‘step-wise method’, which allows for multiple mutations per marker by summing a genetic distance between two sets of Y-STR alleles [1]. While pair-wise TMRCA calculation is trivial, these methods are not readily expandable to multiple people, and do not account well for back mutations (where mutated STRs revert to the ancestral allele), parallel mutations (when two lineages mutate to the same allele), or multi-step mutations (where more than one repeat is added or subtracted from the STR length). Instead, the mutation-rate-normalised variance of Y-STRs can be used to estimate the coalescence age of a larger haplogroup or population [2]. However, this treats clades as homogeneous units, hence does not properly account for genetic drift within populations. A tree-based solution is needed to overcome this problem, such as batwing [3]. However, all these methods suffer from saturation problems where the number of Y-STR mutations does not become cleanly countable. The average squared deviation method (e.g., [4]) improves on this, but none of these methods natively allow for phylogenic ordering from Y-SNPs.

A tree-based method using Y-SNPs has been developed [5], reliant on counting mutations between nodes in the phylogenic tree, then averaging sub-clades together to calculate the TMRCA of the parent clade. While a major innovation in TMRCA calculation, it incurs problems with causality (where parent clades can become younger than child clades), does not optimally weight the influence of sub-clades of differing sizes, and does not treat uncertainty in a fully Poissonian way. An improved, Bayesian approach has been developed into the beast code [6], with the added advantages of automating the tree topology using Markov-Chain Monte-Carlo (MCMC) sampling, and allowing a relaxed mutation rate, but this requires considerable computing resources to run on today’s large genetic databases.

The demands of genetic genealogy are exacting. Customers desire the precise generation in which two people are related, namely uncertainties of ∼±30 years over 200 years of history. More realistic expectations might be to identify a surname’s cultural origin (∼±250 years over 800 years), or to link pre-historic migrations with specific haplogroups (∼±500 years over 5000 years). This accuracy requires a new method that combines the Y-SNP and Y-STR molecular clocks, and takes into account other available evidence (e.g., ancient DNA, proven paper genealogies, relatedness through autosomal DNA, etc.). Here, I provide a mathematical basis to merge these methods, improving on the accuracy of each. I also discuss the potential second-order effects that will need to be addressed before any large-scale, practical application of this method can be made. The simple implementation of this method used in the examples below is available at https://github.com/iain-mcdonald/TMRCA.

## 2. Materials and Methods

### 2.1. Driving Mathematical Principle

The age between any two connected nodes on the Y-DNA phylogenic tree can be expressed as the combination of lines of evidence, each one represented as a probability distribution function (PDF), P(t|e): the probability (*P*) of a relationship at a time in the past (*t*) given evidence (*e*). That evidence can be broken down into *n* pieces, such that the combined probability is simply the multiplication of all probabilities involved:

(1) $$P\left(t|e\right)=k\prod_{i=1}^{n}P\left(t|e_{i}\right).$$

The normalisation constant, *k*, is derived *a posteriori* such that $\int_{0}^{\infty }P\left(t|e\right)dt=1$. Broadly speaking, the evidence, *e* falls into three categories: Y-SNPs including ancient DNA (Section 2.2), Y-STRs (Section 2.5) and historical information (Section 2.6). However, any piece of evidence can be included, provided it can be accurately represented in this form.

We make the simplifying assumption that the exact point in time we are trying to identify is the birth of the common ancestor of all men within a Y-DNA haplogroup, which we can assume is any grouping of two or more related testers. For small groups, perhaps up to ∼10 individuals, the dominant uncertainties are Poisson uncertainties from the small number of mutations, and the method by which mutations are counted; for larger, older group, the accuracy of the mutation rates dominates; while for larger but recent groups, a number of second-order statistical and genetic effects become important.

### 2.2. Pdfs from ‘Next-Generation’ Sequencing Tests

#### 2.2.1. Guiding Principles for TMRCA Calculation from SNP Counting

A sequencing test reads a subset of the MSY and identifies mutations compared to a reference sequence, which may need correcting to an ancestral reference sequence, rather than the standard human genome (where SNPs ancestral to R1b-U152 are mostly derived). The MSY subset can be defined as a set of *b* callable loci, where Y-SNPs can securely be detected (the test’s “coverage”). Let us say we count *m* novel SNPs within these callable loci. Given *N* tests, the coalesence age of the haplogroup is given to first order by

(2) $$t_{\mathrm{SNP}}\approx \frac{1}{\mu_{\mathrm{SNP}}}\sum_{n=1}^{N}\frac{m_{n}}{b_{n}},$$

The mutation rate, $\mu_{\mathrm{SNP}}∼8\times 10^{-10}$ SNPs per base pair per year (Appendix A.4) must be definable over all *b* loci, so further restriction of *b* may be needed to account for this. Hence, $1/b\mu_{\mathrm{SNP}}$ is the effective temporal resolution of a specific test. Most commercial tests have b∼15 million base pairs (Mbp), hence $1/b\mu_{\mathrm{SNP}}\approx 83$ years per SNP.

This calculation allows us to create P(t|m), the probability that *t* years have passed since the MRCA, given *m* observed mutations. The dominant uncertainties in this quantity derive from the uncertainty in the mutation rate and Poisson noise from the small number of mutations. Hence, we can specify that, for one test,

(3) $$P\left(t|m\right)=\mathrm{Poisson}\left(m,tb\mu \right)=\frac{{\left(tb\mu \right)}^{m}\exp\left(-tb\mu \right)}{m!}.$$

The analysis here is applied to SNPs, but a similar approach can be used for other types of mutation recovered from sequencing tests (e.g., insertions, deletions, complex variants), provided a mutation rate for them can be accurately defined.

#### 2.2.2. Dealing with Uncertainty in μ for Multiple Tests

The uncertainty in the mutation rate ($\sigma_{\mu }$) essentially provides an unknown scaling factor to the entire phylogenic tree, making it overall longer or shorter but without adjusting the spacing between branches. This scaling factor only needs to be applied when the conversion from number of mutations to physical time is performed. Since the uncertainty in the mutation rate is typically small compared to the overall uncertainty, it may be advantageous to compute the entire tree in terms of number of nominal mutation timescales, then convert to a physical time afterwards. An alternative method is to apply the uncertainty in μ as a multiplicative broadening to each node of the tree.

Which of these methods is most appropriate depends on the length of the tree and whether data beyond Y-SNPs are being treated. If the tree is dominated by recent nodes (so that dominant uncertainty remains Poisson broadening), and/or if significant constraints are anticipated from other information (e.g., historical information, Y-STRs or ancient DNA), then it may be advantageous to treat the uncertainty in μ as each node is computed.

#### 2.2.3. Defining *b* and *m* for Multiple Tests

In order for Equation (3) to be valid, the mutations (*m*) should be a subset of callable loci (*b*). However, differing coverage between tests makes it impossible to determine with accuracy whether some SNPs are common to a particular haplogroup, or an upstream or downstream haplogroup. Often, placement can still be reliably made using lower-quality calls from the haplogroup’s other tests. However, in many cases, there are still too few reads to be used.

The extreme solutions is to restrict the coverage (*b*) of each test to the set of callable loci common to all the tests in the haplogroup, but this results in a needlessly declining coverage as one progresses up the haplotree. An alternative is to use the combined coverage where at least one test contains a callable locus, and use Markov chains to explore each possibility, placing the SNPs without certain placement in different haplogroups in each chain, and averaging the probabilities of those chains. This method can be computationally very intensive, but can yield a much more accurate solution where coverage differs significantly.

A compromise between the two methods is to take the subset of base pairs where a callable locus exists in at least two samples in two different immediate sub-clades ($\bar{b}$). If the phylogenic tree is organised such that SNPs with uncertain placement are associated with haplogroups as close to present day as possible, SNPs not falling into the coverage $\bar{b}$ (therefore imprecisely placed on the phylogenic tree) can then be ignored in the calculation, negating the need for Markov chains while utilising the coverage of each test effectively. Generalising to *N* tests or child clades defining a haplogroup, $\bar{b}$ can be mathematically defined as the intersection of coverage for any two of those tests or child clades, or the union of these intersections:

(4) $$\bar{b}=\overset{N}{\underset{i=1}{⋃}}\overset{N}{\underset{\left(j=1,j\neq i\right)}{⋃}}b_{i}\cap b_{j}.$$

Here, $b_{i}$ or $b_{j}$ is the intersection of *all* the tests from sub-clade *i* or *j*, respectively, since only one person from each sub-clade needs to be called on a given base pair. This is statistically unbiased unless the occurrence of a mutation strongly influences whether a base pair has coverage. If the occurrence of a mutation does affect, e.g., the mapping quality of reads, then this will tend to miss out mutated regions and bias the resulting TMRCA towards younger ages.

A significant problem in performing this analysis is the removal and treatment of errant SNPs, which is discussed in Appendixes Appendix A.2 and Appendix A.3.

#### 2.2.4. Parsing the Phylogenic Tree

Excepting (grand-)father–son pairs, individual tests represent distinct lineages, so can effectively be treated as different lines of evidence within Equation (1). For each test or sub-clade (k=1,⋯,N) within a haplogroup, we can count the mutations ${\bar{m}}_{k}$ that occur within coverage filter $\bar{b}$. Equation (3) then becomes

(5) $$P\left(t|m\right)=\frac{\prod_{k=1}^{N}\mathrm{Poisson}\left({\bar{m}}_{k},t\bar{b}\mu \right)}{\int_{0}^{\infty }\prod_{k=1}^{N}\mathrm{Poisson}\left({\bar{m}}_{k},t\bar{b}\mu \right)dt}.$$

However, where the haplogroup contains sub-clades, the existing TMRCA of the sub-clade and its PDF need to be taken into account. Naïvely, the age of a parent clade, $t_{p}$ is simply the age of each child clade, $t_{c}$, plus the time between the child and parent clades’ TMRCAs, $t_{c\to p}$, averaged over the set of sub-clades (k=1,⋯,N):

(6) $$t_{p}=\frac{\sum_{k=1}^{N}t_{c}+t_{c\to p}}{N}.$$

This is the calculation employed on the YFull database (http://www.yfull.com, accessed 3 June 2021) [5]. However, this calculation gives incorrect weight to different child clades, as it will equally weight a high-precision clade with many testers and a low-precision clade with only one tester.

Since both $t_{c}$ and $t_{c\to p}$ are probability distributions, represented by Equation (5), we can revise Equation (6) to be probabilistic by performing the convolution of the two PDFs describing the age of the child clade ($P(t_{c}|m_{c})$) and the time between child and parent clade ($P(t_{c\to p}|m_{c\to p})$), such that:

(7) $$P\left(t_{p}|m_{c},m_{c\to p}\right)=P\left(t_{c}|m_{c}\right)*P\left(t_{c\to p}|m_{c\to p}\right).$$

Note that the convolution is only valid for times $t_{p}>0$, as the parent must be older than the child: the Poisson distribution ensures $P(t_{p}<0)$ is always zero.

Based on this formalism, the combination of many sub-clades (*k*) to yield the age of a parent becomes:

(8) $$\begin{array}{ccc} P(t_{p}|k) & = & \frac{\prod_{k=1}^{N}p_{k}\left(t\right)}{\int_{0}^{\infty }\prod_{k=1}^{N}p_{k}\left(t\right)dt}\\ & & \;\mathrm{where}\;\left\{\begin{array}{cc} p_{k}\left(t\right)=P\left(t_{c}|m_{c}\right)*P\left(t_{c\to p}|m_{c\to p}\right) & \mathrm{if}\;a\;\mathrm{sub}-\mathrm{clade},\;\mathrm{or}\\ p_{k}\left(t\right)=P\left(t_{c}|m_{c}\right)*P\left(t_{b}\right) & \mathrm{if}\;a\;\sin\mathrm{gle}\;\mathrm{tester}. \end{array}\right. \end{array}$$

Here, $P(t_{b})$ is a PDF describing the birth date of the tester involved. This will typically be around 1950 CE (Appendix A.1) but may be different, e.g., for ancient DNA samples.

Using this method, a set of TMRCAs can be progressively indexed to nodes of the phylogenic tree, starting from the lowest (most-recent) nodes, and building up to the head node of the tree.

### 2.3. Treating Causality: Constraint from the Parent Clade

Random processes mean some clades will have statistically too many or too few SNPs. When few mutations separate parent clades from child clades, Equation (6) can result in causal impossibilities, where children are older than their parents.

There are three potential causes of these causality problems. First, the stochastic production process of SNPs, which should average out as clades are merged together. Second, changes in the underlying mutation rate of a haplogroup, which we ignore here (Appendix A.4). Third, during periods of rapid population growth, mutations occurring within the first few generations of a haplogroup’s MRCA will be present in proportionally more of the haplogroup, hence will go on to define the haplogroup’s larger child clades, and leading to an imbalance whereby larger child clades receive statistically more mutations than smaller child clades (Appendix A.4.1).

One could apply a sub-clade-specific mutation rate, and scale the TMRCAs of the entire child clade by a specific correction factor. This is appropriate if the mutation rate of haplogroup physically changes, but not if the mutation rate is dictated by random processes: random processes operate more strongly on the upper regions of the phylogenic tree, where there are fewer branches at a given time and random effects can have proportionally more impact.

Instead, we can use the TMRCA of the parent clade as a *semi*-independent constraint. By reversing Equation (7), we can derive the TMRCA of the child clade from its parent, given the PDF for the parent clade’s TMRCA ($P(t_{p})$) and the number of mutations separating parent and child ($m_{c\to p}$):

(9) $$P\left(t_{c}|\left(P\left(t_{p}\right),m_{c\to p}\right)\right)=P\left(t_{p}\right)*P\left(t_{c\to p}|-m_{c\to p}\right).$$

Here, the coverage is the child clade’s $\bar{b}$. The result is that clades immediately following a period of growth have their ages equalised, in line with their parent, but without substantial effect on clades closer to the present.

There is an element of circular logic in this problem, in that the TMRCA of a parent clade is already derived from those of its children. Hence, Equation (9) is only valid if the child clade contributes negligibly to the TMRCA of the parent. A modification of Equation (9) is possible, replacing the convolution with an addition,

(10) $$P\left(t_{c}|\left(P\left(t_{p}\right),m_{c\to p}\right)\right)=P\left(t_{p}\right)+P\left(t_{c\to p}|-m_{c\to p}\right).$$

If the child clade wholly defines the TMRCA of the parent, and Equation (10) is used as an independent constraint in Equation (1), it will be given equal weight to Equation (9), thus provide half the nominal correction. However, it can lead to negative TMRCAs (over-correction) for recent clades. In theory, an iterative process could be used to provide an exact solution. However, a good compromise can come from an average of Equations (9) and (10), weighted according to the contribution of the child clade to the total TMRCA (e.g., by the square root of the number of kits), with $P(t_{c}<0)$ set to zero.

### 2.4. Inclusion of Ancient DNA

Theoretically, if an ancient DNA sample is called for novel variants, it can be treated like any other test, and $P(t_{b})$ set to the probability distribution of the calibrated ${}^{14}$C date, or the archaeological period, as appropriate.

More usually, poor DNA recovery does not allow novel variants to be called. Even if they can be called, deamination and other DNA damage can cast doubt on the validity of novel variants in ancient DNA. This means ancient DNA can often only provide a lower limit to the age of any particular SNP and its associated haplogroup. $P(t_{b})$ can then be represented as the cumulative distribution of the calibrated ${}^{14}$C date (or some measure of the cultural frequency if no carbon date is available). Assuming one is calibrating ages to the birth years of testers, the age of the ancient individual at death should technically be taken off any ${}^{14}$C date, although in practice this is a small change.

Relatedly, since ancient DNA samples have ages defined by calendar dates, the uncertainty in the SNP mutation rate needs to be taken into account before the ancient DNA can be included (Section 2.2.2). This can be done on a node-by-node basis as the tree is calculated, with consequent reduction in the precision of the Y-SNP-based tree.

In the “cleanest” cases, ancient DNA can be unambiguously called for all the individual SNPs associated with a particular haplogroup: as a basal member of that haplogroup, its $P(t_{b})$ can then be applied directly as a constraint to the TMRCA. More often, only a partial set of these SNPs will be callable, due to the comparatively poor recovery of the ancient DNA sample.

Otherwise, one of two methods could be applied. Firstly, an intermediate node can be created between two haplogroups, separating out the positive and negative SNPs into younger and older haplogroups, respectively. A Markov-chain approach may then be needed to deal with uncalled or ambiguously called SNPs (cf., Section 2.2.3). Alternatively, a new node can be branched from the parent haplogroup, duplicating the SNPs that are positive in the ancient DNA sample into a brother clade. This has the disadvantage of losing a small amount of accuracy in the downstream haplogroups, but results in a single calculation where the ancient DNA sample’s effective coverage can be used directly.

Often, calls for SNPs in ancient DNA, and the associated coverage of the ancient DNA test, are based on lower quality standards than DNA tests on living people. Hence, some remnant uncertainty exists as to whether a SNP is truly positive or negative. In this case, applying an offset like Equation (24), or applying a weighted Markov chain, would allow this probability to be dealt with.

### 2.5. PDFs from Y-STR Alleles

#### 2.5.1. Guiding Principles

The longer history and lower price of consumer Y-STR testing means many more Y-STR results exist than NGS test results. In theory, the greater abundance and faster mutation rate of Y-STRs allow much more precise TMRCAs to be calculated than for Y-SNPs. The molecular clock for an individual Y-STR follows exactly the same principles as the Y-SNP molecular clock. Specifically, for a particular Y-STR (*s*) with mutation rate $\mu_{s}$, the number of mutations experienced over time can be expressed as

(11) $$m_{s}=t\mu_{s}.$$

Hence, the probability of receiving $m_{s}$ mutations over a specific time *t* is the product distribution of the appropriate Poisson distribution and the PDF describing the uncertainty in $\mu_{s}$:

(12) $$P\left(t|m_{s}\right)=\mathrm{Poisson}\left(m_{s},t\mu_{s}\right)⊗P\left(\mu_{s}\right).$$

Since $P(\mu_{s})$ is different for each STR, it can prove more practical to deal with this uncertainty at this initial stage of the calculation. The combination of s=1..N Y-STRs can then be calculated as

(13) $$P\left(t|m_{\mathrm{STRs}}\right)=\prod_{s=1}^{N}P\left(t|m_{s}\right).$$

The mutation rates for Y-STRs are (individually and collectively) more uncertain than Y-SNPs, and there is some evidence that mutation rate varies with STR length. There may also be a long-term selection component that means mutations are not truly random. These factors are discussed in Appendix A.5. However, the principal problem with Y-STRs is that they suffer from convergent mutations. These include hidden mutations, where an STR mutates, then mutates back to its original length in a later generation; ‘multi-step’ mutations, which add or delete more than one repeat, and cannot be differentiated from two unique mutations; and parallel mutations, where two lines independently acquire the same mutation. Consequently, we do not observe $m_{s}$, but rather the genetic distance of each STR ($g_{s}$), so we require the additional conversion $P(m_{s}|g_{s})$: the probably of obtaining genetic distance $g_{s}$ given $m_{s}$ mutations. We also need to account for the uncertainty in $\mu_{s}$ ($\sigma_{\mu ,s}$). Hence Equation (13) becomes

(14) $$P\left(t|g\right)=\prod_{s=1}^{N}\sum_{m_{s}=0}^{\infty }P\left(t|m_{s}\right)P\left(g_{s}|m_{s}\right).$$

These convergent mutations mean placement in a phylogenic tree from Y-STR testing alone can be ambiguous. Hence, for this discussion, we will concentrate on individuals where a haplogroup has already been robustly identified from Y-SNP results. In cases where a haplogroup has not been identified, computing P(t|g) using the genetic distance from two modern tests should give twice the TMRCA, while accounting for convergent mutations.

Equation (14) requires a count of mutations from the haplogroup’s ancestral Y-STR motif, so this first needs to be established (Section 2.5.2). We then need $P(m_{s}|g_{s})$ (Section 2.5.3). Finally, we need the relevant mutation rates (Appendix A.5). Mutation rates for Y-STRs are traditionally given in units of per generation, rather than per year: Appendix A.5.1 describes the calibration needed to convert generations to years.

Once obtained, $P(t|m_{\mathrm{STRs}})$ and $P(t|m_{\mathrm{SNPs}})$ for each tester can each be used as independent estimates in Equation (1), or used separately to investigate the long-term mutation properties of STRs. Clades can then be combined using an identical strategy to that described in Section 2.2 for SNP-based ages from NGS tests. This allows an identical tree to be built up, with nodes (haplogroups) on the tree defined by Y-SNPs, but with ages derived from Y-STRs.

#### 2.5.2. Determination of Ancestral Y-STR Motifs

Constructing the ancestral Y-STR motif for a clade is eased by an existing phylogenic tree based on Y-SNPs. Nevertheless, it can rarely be performed with absolute accuracy across large datasets, and will only work adequately with mostly complete datasets. Hence, some approximations and/or use of Markov chains may be required to accurately reproduce TMRCAs using this method, and it is really most useful in examining particularly young (surname-era) clades or the rapid expansion of a set of well-populated older clades. Null or ambiguous entries for the ancestral alleles of Y-STRs are statistically the most likely to be those where a mutation has occurred, thus it is possible for TMRCAs to become underestimated if mutations are missed.

Batwing [3] and other programmes address the issue of creating ancestral Y-STR motifs, and the reader is referred to those codes for detailed guidance. However, creating an ancestral set of Y-STR alleles for each node in the phylogenic tree can also follow similar principles to creating a TMRCA for each node in the tree from SNPs: an iterative approach can be used, first passing up the tree computing a modal of downstream sub-clades, then passing down the tree using the allele of the parent clade to fill in missing data. The parent allele often also provides a good approximation in ambiguous cases, such as where the parent allele does not match any child clade, or where the alleles of child clades are multi-modal.

#### 2.5.3. Terminology

The following terminology is used below: + for a single increase in Y-STR length (e.g., 12→13) and − for a single decrease (e.g., 12→11); ${+}^{2}$ for a multi-step increase by two repeats (e.g., 12→14); and similarly ${-}^{2}$, ${+}^{3}$, ${-}^{3}$, etc. A change in length from 12 to 14 units can be two mutations, [+,+], or a single multi-step mutation of $\left[{+}^{2}\right]$, or as some arbitrarily complex set of forward and back mutations, e.g., $\left[{+}^{4},-,-\right]$.

Each mutation has a probability of occurring, ω, so that we can define $\omega_{+}$, $\omega_{-}$, $\omega_{+2}$, $\omega_{-2}$, etc. Similarly, we can define $\omega_{\pm }=\omega_{+}+\omega_{-}$, and similarly for $\omega_{\pm 2}$, $\omega_{\pm 3}$, etc. These generally poorly quantified: reference [7] identifies eight multi-step mutations out of 620 total, suggesting $\sum_{n=2}^{\infty }\omega_{\pm n}\approx 0.0129$ (95% c.i. 0.0060–0.0263). The majority of these are two-step mutations, suggesting $\omega_{\pm 2}\approx 0.0097$, and $\omega_{\pm 3}\approx \omega_{\pm 4}\approx 0.0016$. Ref. [8] similarly identifies 30 multi-step mutations out of 787 total, finding $\sum_{n=2}^{\infty }\omega_{\pm n}=0.038$ (95% c.i. 0.026–0.054), with $\omega_{\pm 2}\approx 0.032$, $\omega_{\pm 3}\approx 0.004$ and $\omega_{\pm 4}\approx \omega_{\pm 5}\approx 0.001$. The difference likely reflects the individual STRs typed in the two studies (respectively, 94 and 186 Y-STRs were tested). Here, we adopt multi-step frequencies from [8], as they better match earlier studies, e.g., [9].

Among both single and multi-step mutations, literature is divided regarding whether there a bias toward gaining Y-STRs repeats in single-step mutations, and losing Y-STR repeats in multi-step mutations ($\omega_{+1}/\omega_{\pm 1}=0.5736$ (95% c.i. 0.5296–0.6166, implying $\omega_{-1}/\omega_{\pm 1}=0.4264$ [7]), or vice versa ($\omega_{+1}/\omega_{\pm 1}=0.463$; 95% c.i. 0.403–0.463 [8]). Similarly, whether the overall length of STRs is changing over time is uncertain (e.g., see [10,11,12,13], versus [8,14]). The tension between these results may reflect asymmetric mutation probabilities in individual Y-STR loci ([15]; also [8], their Figure 3), with a probable anti-correlation of $\omega_{+1}/\omega_{\pm 1}$ with mutation length [4]. Consequently, we retain the mathematical possibility that $w_{+}\neq w_{-}$, but assume them to be equal for the purposes of calculation, namely

(15) $$w_{+}=\sum_{n=1}^{\infty }\omega_{+n}=0.5;\;w_{-}=\sum_{n=1}^{\infty }\omega_{-n}=0.5$$

with $\omega_{\pm 1,2,3}=0.962$, 0.032 and 0.004. Values of $\omega_{\pm \geq 4}$ are fairly uncertain but can have an important impact on TMRCA calculations if such large an insertion or deletion occurs. In the calculations below, we decrease the frequency of occurrence by a factor of $\sqrt{10}$ for every additional repeat unit after $\omega_{\pm 3}$ and slightly adjust $\omega_{\pm 1}$ to 0.96217 to ensure $w_{+}+w_{-}=1$.

#### 2.5.4. Mapping Mutations from Genetic Distance: General Formulae

Equation (14) introduces the mapping $P(g_{s}|m_{s})$. For this section, we drop the subscript notation and refer to the single-STR case of P(g|m).

For low $m_{s}$, the translation is simple. No mutations must always lead to g=0, hence P(g=0|m=0)=1 and P(g>0|m=0)=0. For a single mutation, we have P(g=0|m=1)=0 (one mutation must always leave a non-zero genetic distance). However, $P\left(g\;=\;n|m\;=\;1\right)=\omega_{n}$.

For m>1, the direction of mutation becomes important, as in half of cases the subsequent mutation will cancel out the first one. Hence, $P\left(g\;=\;0|m\;=\;2\right)=\sum_{n=1}^{\infty }\omega_{\pm n}^{2}/2$, while $P\left(g\;=\;\pm 1|m\;=\;2\right)=\sum_{n=-\infty }^{\infty }\omega_{\mp n}\omega_{\pm n+1}$. As *m* increases, the permutations that can create *g* become more complex.

Generalising to arbitrary *m*, any set of genetic mutations can be thought of as a set of $k_{+}$ positive and $k_{-}$ negative single-step mutations, which can be considered as randomly selected from the binomial distribution,

(16) Cmk+=m!(m−k+)!k+!.

The resulting probability of obtaining $k_{+}$ from *m* is the corresponding binomial mass probability distribution:

(17) P(k+|m)=Cmk+w+k+w−k−.

We can now consider replacing some of the *m* mutations with an optional, arbitrary set of $Q_{n+}$ positive and $Q_{n-}$ negative multi-step mutations of step size *n*. We can then define the sum of *extra* repeats caused by the multi-step nature of these mutations ($\varepsilon_{+}$, $\varepsilon_{-}$) as:

(18) $$\varepsilon_{+}=\sum_{n=2}^{\infty }\left(Q_{n+}-1\right);\;\varepsilon_{-}=\sum_{n=2}^{\infty }\left(Q_{n-}-1\right),$$

meaning that the total number of positive and negative mutations ($k_{+},k_{-}$) needed to obtain *g* from *m* is:

(19) $$k_{+}=\frac{m+g-\varepsilon_{+}+\varepsilon_{-}}{2};\;k_{-}=\frac{m-g+\varepsilon_{+}-\varepsilon_{-}}{2}.$$

The probability of obtaining a particular number of multi-step mutations ($Q_{n+}$) of any step size *n* from $k_{+}$ positive mutations can be nested within Equation (17) to become:

(20) P(Qn+|m)=Cmk+w+k+w−k−∏n=2∞Ck+Qn+ω+nw+Qn+1−ω+nw+k+−Qn+∏n=2∞Ck−Qn−ω−nw−Qn−1−ω−nw−k−−Qn−ifk+,k−∈Zotherwise0.

Naturally, a similar equation exists for $P(Q_{n-}|m)$. For $w_{+}=w_{-}=0.5$, these reduce to:

(21) P(Qn|m)=Cmk+2m∏n=2∞Ck+Qn+Ck−Qn−ω±nQn++Qn−(1−ω±n)m−Qn++Qn−ifk+,k−∈Zotherwise0.

Having calculated the probability for a particular $Q_{n}$, we can sum the contributions for all possible $Q_{n}$. Mathematically, we can treat each type of multi-step mutation (e.g., two-step, three-step, ...) as a single calculation, and frame the calculation in terms of a set of functions $f_{r}$, which contain factors with *r* different kinds of multi-step mutations:

(22) $$\begin{array}{ccc} P(g|m) & = & \sum_{r=0}^{\infty }f_{r},\;\mathrm{where}:\\ f_{0} & = & P(Q=0|m)\\ f_{1} & = & \sum_{n=2}^{\infty }\sum_{i=1}^{\infty }P\left(Q_{n+}=i|m\right)+P\left(Q_{n-}=i|m\right)\\ f_{2} & = & \sum_{n=2}^{\infty }\sum_{n^{\prime }=2}^{\infty }\sum_{i=1}^{\infty }P\left(Q_{n+}\;=\;i,Q_{n^{\prime }-}\;=\;i|m\right)+\\ & & \;\;\sum_{j=i+1}^{\infty }P\left(Q_{n+}\;=\;i,Q_{n^{\prime }-}\;=\;j|m\right)+P\left(Q_{n-}\;=\;i,Q_{n^{\prime }+}\;=\;j|m\right)\\ & & \mathrm{etc}. \end{array}$$

In other words, $f_{0}$ is any set of mutations containing no multi-step mutations, $f_{1}$ has one type of multi-step mutation (positive or negative), $f_{2}$ has two types of multi-step mutations (two positive, two negative or one of each). While this sum extends to an arbitrary number of multi-step mutations of arbitrary complexity, in practice, only a few of these are important. Mixed positive and negative multi-step mutations are accounted for here, but can generally be neglected, as the probability of getting these is always much lower than the single-step alternative (e.g., the weight for $\left[{+}^{2},{-}^{2}\right]$ is ${\left(\omega_{\pm 2}/\omega_{\pm 1}\right)}^{2}\approx 1/900$th that of [+,−]), meaning terms $f_{3}$ and higher, and the *j* summand in $f_{2}$ can usually be ignored. Table 1 lists computed probabilities of P(g|m) for the values of ω listed in Section 2.5.3.

This then lets us solve Equation (14). Assuming Gaussian uncertainties of $\sigma_{\mu }$ for $\mu_{s}$, Equation (14) becomes

(23) $$\begin{array}{ccc} P(t|g_{\mathrm{STRs}}) & = & \prod_{s=0}^{S}\int_{\mu^{\prime }=0}^{\infty }\left(\sum_{m_{s}=0}^{\infty }\left(\;\mathrm{Poisson}\left(t\mu_{s}^{\prime },m_{s}\right)P\left(g_{s}|m_{s}\right)\right)\right.\\ & & \left.\cdot \frac{1}{\sqrt{2\pi }\sigma_{\mu_{s}}}\exp\left(-\frac{{\left(\mu_{s}-\mu_{s}^{\prime }\right)}^{2}}{2\sigma_{\mu_{s}}^{2}}\right)\right)d\mu_{s}^{\prime }. \end{array}$$

The problem is further complicated if multi-copy markers are included. Here, we cannot always be conclusive about the genetic distance without knowing which copy of the marker is which. For example, a mutation of DYS464 from an allele of 15-16-16-17 to 14-15-16-17 could represent one mutation (16→14) or two mutations (15→14 and 16→15). Recombinative loss of heterozygosity (recLOH) events in multi-copy markers also present as multi-step mutations. Hence, if these markers are to be included in the TMRCA calculation at all, it is generally easier to determine whether or not there has been *any* mutation of the marker, and apply P(t|g=0) or the sum of P(t|Σg>0) as appropriate. Note that this is an approximation, as it does not properly treat parallel mutations of different copies (e.g., 14-15-16-17 and 14-16-15-17 will be read identically), but it may be useful to implement in the case of rapidly mutating multi-copy markers like CDY, particularly for short timescales.

### 2.6. Historical and Ancillary Information

Ancillary information can be combined with Y-DNA tests to improve the TMRCA calculation, creating an informed prior on P(t). Such data include paper genealogies, shared surnames, autosomal DNA tests, and historical population sizes. The mathematical properties of this data can be hard to quantify (e.g., when determining how trustworthy or error-free a piece of information is). Hence, care must be taken not to impart circular logic to prove or disprove a hypothesis, especially given the potentially recursive nature of the calculations.

#### 2.6.1. Paper Genealogies

Paper genealogies represent the simplest relationship constraint, as time is already the dependent variable. By genealogically anchoring a haplogroup to a specific person, the coalesence ages of the surrounding haplogroups can be improved. Typically, genealogies fall into two categories: either two haplotyped individuals have a known common ancestor, or where they show no common ancestor after a certain date (normally their oldest proven ancestor).

Precisely known common ancestors can be represented with a δ function as P(t), creating an infinitely narrow peak at the age of the common ancestor (*T*). In a grid computation, it is important to make sure the δ function is actually sampled. A smoother function can be applied if a common ancestor’s birth date is not known exactly, e.g., a Gaussian function for an uncertain date, or a boxcar function for a period bracketed by two records, or a log-normal function to depict a time of birth based on a historical record like a marriage. Such functions can be combined arbitrarily.

#### 2.6.2. Shared Surnames

In general, two individuals within a sufficiently small haplogroup are unlikely to share a surname unless they are closely related. In this case, “unlikely” can be defined as the chance of randomly matching two people with that surname in the wider haplogroup ($\psi_{2}$) and “closely related” depending on when male-inherited surnames stablised in the host culture ($T_{\mathrm{surname}}\pm \sigma_{\mathrm{surname}}$). A cumulative Gaussian function can then be used to define a probability:

(24) $$P\left(t\;|\;\mathrm{shared}\;\mathrm{surname}\right)=\psi_{2}+\frac{1-\psi_{2}}{2}\left[1+\mathrm{erf}\left(\frac{t-T_{\mathrm{surname}}}{\sqrt{2}\sigma_{\mathrm{surname}}}\right)\right].$$

Offsets like $\psi_{2}$ can also be used in cases where a MRCA is suspected, but not proven. The difficulty here is in selecting an objective probability from subjective evidence. $T_{\mathrm{surname}}$ varies globally from the last few hundred to ∼1500 years, and the principle can be extended to (e.g.) clan septs sharing a known common origin [16], or historical events like the R1b-L151 Corded Ware culture starburst [17].

Conversely, lack of a shared surname does not necessarily disprove a close relationship. As well as intentional surname changes, non-paternity events commonly result in surname changes. Estimates of the frequency of such changes vary significantly from culture to culture, and time period to time period, but can be approximated to 1–2 per cent per generation on average (e.g., [18]). While a corrective function could be generated, choosing the correct rate may be difficult and highly specific to populations and epochs. Therefore, when surnames are not shared, it may be best to retain a flat-prior probability distribution unless more direct evidence of a non-paternity event or other surname change exists.

#### 2.6.3. Autosomal DNA Tests

Autosomal DNA testing is extremely common among commercial Y-DNA testers. Shared autosomal DNA can usefully constrain a relationship to between one and a few generations in the past. Equally, a lack of an autosomal DNA relationship normally indicates a relationship at least ∼5–7 generations ago. The relevant probability functions can be approximated as a log–normal and cumulative Gaussian functions, respectively, but the exact constants used in this calculation will depend on the details of the autosomal tests involved and the level of endogamy expected in the tests.

#### 2.6.4. Historical Population Sizes

Historical population sizes play an important, but normally overlooked role in calculating TMRCAs. While intentional testing of close cousins is common among the genetic genealogy community, these are almost universally known cousins. Hence, if a shared ancestor is not present, it can normally be assumed that any test will be genetically matched against random members of a wider population. Thus, a person is equally likely to match any given close cousin as a distant one. However, a person will typically have many more distant cousins than close cousins. This heavily skews the probability towards older TMRCAs. Consequently, adopting an appropriate multiplicative prior (e.g., statistical estimates of number of cousins of the $n^{th}$ degree) may be important in obtaining accurate TMRCAs, especially in historic times.

This prior can be determined using the male-specific net reproduction rate (NRR): the average number of cousins of a particular degree a person has should increase every generation by the NRR, namely:

(25) $$P\left(t\;|\;\mathrm{NRR}\right)={\mathrm{NRR}}^{t/G},$$

where *G* is average number of years per generation (Appendix A.5.1). This prior increases the TMRCA by of order the average NRR, e.g., if NRR = 1.3, the TMRCA increases by ∼30%. Since NRR can vary significantly by population and throughout time, it may be necessary to provide population-specific priors. Furthermore, since there is considerable variation in the NRR for individual families, the accuracy and applicability of this correction has the potential to dominate the uncertainty budget among very-well-tested historical families, and set the fundamental limit to the accuracy of TMRCA calculations.

This prior is most relevant in recent centuries, where the fractional uncertainty in TMRCA and NRR are both large (e.g., [19]). It becomes a relatively minor correction in prehistoric times, where Poisson uncertainties tend to dominate (for example, from a population of ∼20,000 living 130,000 years ago [20], with an average generation length of 35 years, to reach 1.6 × 10${}^{9}$ people by 1900 AD requires an average NRR of ≈1.0012).

### 2.7. Calibration

We now have a series of probabilities from historical records, Y-SNPs including ancient DNA, and Y-STRs, which can all be used as independent evidence in Equation (1) to create a global TMRCA. However, a number of calibrating factors are still required. These are not central to the model, but are required to compute results, hence we discuss them in Appendix A.

## 3. Results

In principle, this method could be applied to the entire human Y-DNA haplotree. However, the computational complexity of such a venture is beyond the scope of this paper. Instead, this discussion focusses on representative examples, designed to be typical of scenarios faced by real-world testers. By randomly generating examples we also gain the advantage of tracing the performance of the mathematical model while acknowledging any hidden or convergent mutations in the test results. A real-world implementation is presented at the end.

In each generated case, we presume that all individuals concerned have taken a modern commercial Y-DNA sequencing test, variations of which are offered by multiple companies, which includes testing of 111 Y-STRs, each of which have a useable mutation rate. In many cases, testers will also have additional Y-STR matches, but the complication of adding them in a statistically robust manner means we ignore them for these examples. Mutual test coverages ($\bar{b}$) are drawn from a normal distribution of 14 million base pairs, with a standard deviation of one million. A constant value of 35 years per generation is used, with a standard deviation of 8 years. The combined Y-SNP mutation rate of Helgason et al. [21] is used. Otherwise, all computed factors are as listed in the Appendix A. It is assumed that the ancestral STR motif is determinable from upstream haplogroups, which is typical in most cases.

### 3.1. DNA Ancestry within Colonial America

The United States of America represents the dominant market for commercial Y-DNA ancestry testing. Consequently, a frequent aim is to identify whether a relationship is before or after their ancestors emigrated from the Old World to the New World. This provides the short-timescale limit of most Y-DNA testing applications, as testing within the last six generations or so is better accomplished using autosomal DNA testing.

**Figure 1:**
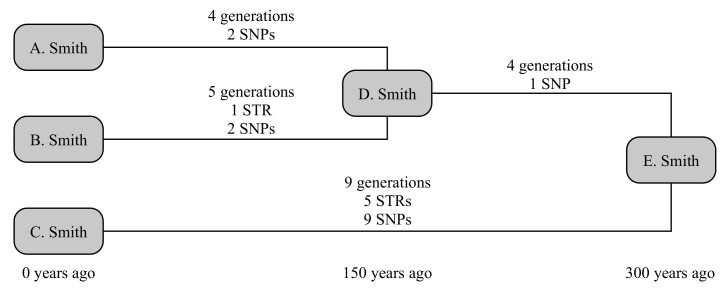
A schematic representation of the example given in Section 3.1.

The example below uses three testers from the Smith family, as illustrated in Figure 1. A. Smith and B. Smith have a common ancestor, D. Smith, who lived 150 years ago. They share a common ancestor with the third tester C. Smith in E. Smith, who lived a further 150 years back. The older relationship is not known to the testers; and we will consider both the scenarios where the younger relationship is and is not known. This gives four times to calculate: D → A, D → B, which are known in the second scenario, and E → D and E → C, which are always unknown.

We can now randomly generate mutations associated with each these lines of descent. A randomly generated number of generations is assigned to each line (4, 5, 4 and 9 generations, respectively). STRs are randomly generated on these lines on a per-generation basis, and SNPs on a per-year basis. In this case, A. receives no STR mutations and two private SNPs, while B. receives a mutation on DYS650 and two private SNPs. Their shared ancestry (E → D generates no STR mutations but one SNP that defines the shared haplogroup of A. and B., while C. receives five mutations (to DYS19, DYS576, DYS570, Y-GGAAT-1B07 and DYS434) and nine private SNPs. Consequently, this creates a situation where fewer than the expected number of both SNPs and STRs occur down lines A. and B., while more than expected number occur down line C. Nevertheless, all SNP and STR frequencies are within the bounds of what would be considered statistically normal (namely, 95% probability interval).

Consider the scenario where we know nothing about the testers’ ancestors. This retrieves a broad range of probable dates, with median 122 years and 95% confidence interval (47–255) years for the age of D., while the age of E. is recovered at 405 (249–627) years. If we instead allow the known age of D. (150 years), this reduces the age of E. to 390 (238–614) years: a small but important step towards the correct answer of 300 years. If we instead use the computed age of E. to refine the age of D., we retrieve that D. was born 93 (36–194) years ago, i.e., a result with greater precision, though lower accuracy, but still statistically correct.

This can be compared to traditional SNP- or STR-only methods, which we compute for the scenario where the age of D. is known. Due to above-average number of SNPs generated for C., and the slightly below-average number of STRs generated for A. and B., these calculations give very disparate dates. The SNP-based method of [5] generates an age for D. of 216 (62–370) years, and for E. of 610 (293–927) years. However, their method slightly underestimates the associated errors at these small ages, as these are computed on the basis that the fractional uncertainty is related to the square root of the number of SNPs, not the underlying Poisson distribution: the corresponding Poisson-distribution values for D. and E. would respectively be 183 (63–400) years and 581 (318–961) years. The STR-based method of [1], using infinite alleles, estimates 2 (0–16) generations or 70 (0–560) years for the age of D. Smith, and an average of 8.5 (3.5–17) generations or 298 (123–595) years for E. Smith. These results are summarised in Table 2.

A high relative degree of precision for the age of E. is obtained by [5] (>±57%, or ${}_{-45}^{+65}$% if Poisson errors are introduced), but this method lacks accuracy in the result due to the comparatively large number of SNPs. By comparison, [1] gives an accurate but imprecise answer (${}_{-59}^{+100}$%) in this case. The combination of both STRs and SNPs in this work provides a more precise answer for the age of E. (${}_{-39}^{+57}$%), which retaining overall accuracy.

While still not meeting the exacting needs of genealogists, the overall TMRCA for the unknown ancestor E. is now robustly constrained to within a few hundred years, or a little over a factor of two. If accurate mutation rates were available for the ∼800 Y-STRs recovered by many commercial tests, or if cousins of C. were tested, this would offer the Smith family the opportunity to define their relationship to perhaps within a century or two.

### 3.2. DNA Ancestry within Historical Scotland and Ireland

A significant application of Y-DNA has been to prove and disprove the descent of Scottish and Irish clans from their semi-mythological (or, at least, historically debatable) family trees, stretching back 1000–1500 years. These are some the most extreme examples of a wider desire to find the origins of surnames across Europe, and serve to exemplify the middle TMRCA range of Y-DNA testing applications.

**Figure 2:**
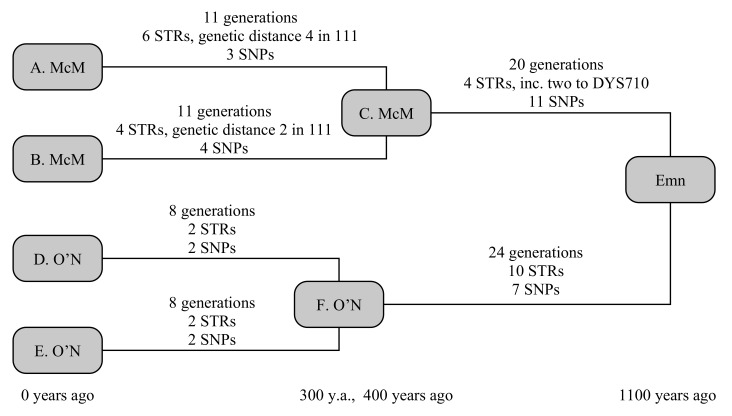
A schematic representation of the example given in Section 3.2.

The example below uses four testers, comprising two chiefly lines from each clan, and shown in Figure 2. The two chieftains from the Scottish Clan McM (A. McM and B. McM) share a common ancestor 400 years ago, but have a known ancestry from primary sources stretching back 800 years. The two chieftains from Irish Clan O’N (D. O’N and E. O’N) share a common ancestor 300 years ago, with a primary-source descendancy of 600 years. Early historical annals claim the most-recent common ancestor, whom we shall call Emn, lived 1100 years ago. We can simulate this to be true, then determine what constraints a real-world observation would be able to place on the TMRCA.

Random generation gives A. McM six STR mutations over 11 generations. Four of these are straight-forward, but the final two include a hidden mutation, comprising a forward and back mutation on the fast-mutating marker CDY, resulting in a net change on only two STRs since his ancestor C. A. McM also receives three private SNPs. Similarly, B. McM accumulates four STR mutations over 11 generations, including a similarly hidden mutation to DYS449, and four private SNPs. Both branches also receive a mutation to the smaller copy of DYS385, but these are in opposite directions, so that the ancestral STR motif is still correctly determined. The O’N clan is somewhat simpler: D. O’N and E. O’N each receive two STR and two SNP mutations over eight generations.

Between Emn and the McM ancestor lie 20 generations, in which four STR mutations and 11 SNP mutations take place. Two of the STR mutations are single-step mutations on DYS710, giving this marker a genetic distance of two. Between Emn and the O’N ancestor lie 24 generations, in which 10 STR mutations and seven SNP mutations occur: none of the STR mutations are in common with the McM line, so none of these 21 mutations are hidden in the calculation.

The TMRCA of the McM and O’N common ancestors are successfully reproduced at 279 (150–470) years and 287 (158–475) years, respectively, with the slightly younger age of the McM common ancestor being due to the two hidden STR mutations. The overall age of Emn is recreated at 1071 (798–1413) years, or 1221 (948–1563) years if the primary-source paper genealogies are accepted, firmly placing the common ancestor in the historical past, and showing the value of including paper-trail genealogies in these calculations.

Using only SNPs, but accepting the primary-source paper genealogies, the [5] model derives an age of 1220 (837–1603) years, correctly placing the older age, but with a wider uncertainty margin. The equivalent Poisson-error model gives 1190 (865–1630) years. Using only STRs under the infinite alleles model [1], and accepting the primary-source paper genealogies, the distance Emn→McM is 10 (3–12) generations and Emn→O’N is 18 (9–24) generations, respectively placing the common ancestor 750 (505–820) and 930 (615–1140) years in the past, or 18 (10–29) generations 980 (700–1365) years for an equally weighted joint distribution. The lower (but still statistically consistent) age from STRs arises from a combination of a statistically low number of STRs generated in both lines, and the two independent mutations of DYS710 occurring in the McM line. These results are summarised in Table 3.

The STR-only model has started to become less accurate in this instance, due to both the back-mutations occurring on some markers, and the assumption of infinite alleles (${}_{-34}^{+23}$%). A step-wise model would provide a small improvement. The SNP-only model is accurate, but when correct errors are adopted becomes somewhat less precise than the model discussed here (${}_{-27}^{+37}$% vs. ${}_{-22}^{+28}$%). The improvement in precision is only fractional, but is sufficient to rule out a century and a half in the possible range, which can be highly significant when trying to place a relationship within historical eras: in this case, the joint model with paper genealogies allows the family to rule out a common ancestor during the Roman or Norman periods of British history at 95% confidence.

### 3.3. DNA Ancestry within Early- or Pre-Historic Europe

British and Irish diaspora dominate Y-DNA testers. A common problem they wish to solve is the time at which their ancestors arrived in the British Isles. TMRCA models cannot address this directly, but insight can often be obtained by identifying the TMRCA between British/Irish individuals and their closest matches of continental European ancestry. Similar questions can be asked of genetic relatedness within any culture lacking written records until the recent past. These represent the longest TMRCAs one might hope to obtain with a joint SNP and STR method.

**Figure 3:**
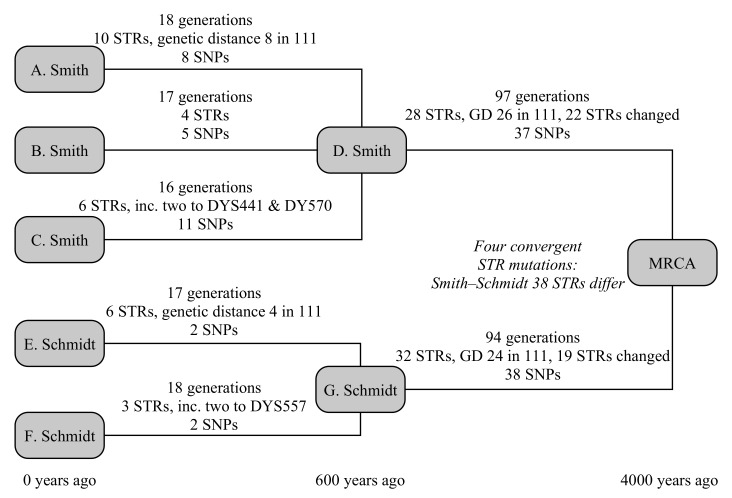
A schematic representation of the example given in Section 3.3.

This example uses five testers, from two different families: three men called Smith from England and two testers called Schmidt from Germany, as illustrated in Figure 3. The testers all know they are not related in the last 250 years, but otherwise have no knowledge of their ancestry. In this case, the common ancestor is set to have lived 4000 years ago, and the Smith and Schmidt families are each internally related 600 years in the past.

The three Smith descendants are modelled to be 18, 16 and 17 generations removed from their common ancestor. In that time, they have built up ten, four and six STR mutations, of which the first Smith has a hidden mutation on DYS578 and the third Smith has two one-step mutations on both DYS441 and DYS570. They have eight, five and 11 SNP mutations, respectively. The Schmidt descendants are 17 and 18 generations removed from their common ancestor, have build up six and three STR mutations (of which the first has a hidden mutation on DYS445, and the second has a two-step mutation on DYS557), and each have eight SNP mutations.

Between the Smith family and the global common ancestor lies 97 generations, and 94 generations for the Schmidt family. They respectively have 28 and 32 STR mutations, plus 37 and 38 SNP mutations. The STR mutations for Smith include a back mutation on DYS504 and two one-step mutations on each of DYS439, CDYb and DYS441, and four one-step mutation on DYS710. The mutations for Schmidt include a three mutations to DYS710 that ultimately lead to an increase in the allele of one, four mutations of CDYb (all in the opposite direction to Smith), and two mutations each on DYS712 and DYS504. Convergent mutations to DYS385b, DYS460, Y-GATA-A10, DYS712 mean that these are hidden from the TMRCA calculation, as the ancestral Y-STR motif derived from a modal or average of the parent clade’s motif and both Smith and Schmidt motifs would yield the derived allele. Consequently, we expect the Y-STR calculation over these timescales to slightly under-estimate the true age of the haplogroup.

The overall TMRCA from the combined SNP+STR model is 3667 (3060–4375) years, correctly predicting the age of the haplogroup within the uncertainty budget (see Table 4). For reference, if only the SNP model is applied for the final step, the resulting age is 3873 (3090–4822) years; if only the STR model is applied, the computed age becomes 3419 (2549–4535) years. The individual ages for the Smith and Schmidt families from the combined model are, respectively, 643 (461–873) years and 519 (328–780) years. Limiting the calculation by enforcing that the Smith/Schmidt TMRCAs are older than the 250-year known ancestry has negligible effect on the final TMRCAs.

The comparison SNP method [5] gives 731 (453–1009) and 710 (384–1036) years, respectively, for the Smith and Schmidt families, however the method of propagating these uncertainties up the tree is not clearly described. Interpreting its use at the YFull website, it is implied that there the uncertainties are not carried forward. In cases where the difference between the TMRCAs of child haplogroups is small compared to the uncertainty (as it is here), this can result in a substantial underestimate of the overall uncertainty budget, particularly if the difference in ages and sizes of the child haplogroups is large. Using the YFull interpretation, the age of the derived haplogroup is 4084 (2757–5411) years: a correct prediction, but a substantially larger uncertainty estimate.

To simplify the STR comparison, we focus on the 3400 years that separate the Smith and Schmidt ancestors from the global common ancestor. The comparison STR method for infinite alleles [1] dramatically underestimates the age, at 56 (40–77) generations, or 1960 (1400–2695) years. This is due to the large number of back mutations (2), convergent mutations (4) and multiple mutations (11) among the STRs (unusually, no multi-step mutations were generated). No existing example of the coded step-wise mutation TMRCA calculation would be found but, at these times, the TMRCA increases approximately linearly with the genetic distance. The existing genetic distance is 35, or 46 including multiple mutations, giving a TMRCA of ∼2600 years.

This test emphasises both the value of combining SNP- and STR-based evidence to create a higher-precision result. However, the most significant benefit here is accounting probabilistically for back, convergent and multiple mutations in the Y-STR-based calculation, and especially accounting for the differing mutation rates of individual Y-STRs, both of which greatly improve the accuracy of the method. Nevertheless, the convergent mutations present in this sparse tree mean that the increased precision that the STRs bring is beginning to be outweighed by the convergent mutations in the tree, which are not accounted for in this model. It may be possible to add a correction for convergent mutations to the TMRCA calculation, at the expense of increased mathematical complexity.

### 3.4. Real-World Example: Royal Stewart Lineages

#### 3.4.1. Data Sources

As a more complex example in the real world, we can explore the descendants of Sir John Stewart of Bonkyll. The Stewart family are a commonly used case in genetic genealogy, as it is known from extensive testing that the SNP S781 formed in Sir John himself (e.g., [22], p. 203; originally J. Wilson, priv. comm.): descendants of his brother (Sir James Stewart, 5th High Steward of Scotland), who form the haplgroup R-Z38845, have the ancestral S781 allele; descendants of two sons of Sir John (Sir James Stewart of Pearston and Sir Alan Stewart of Dreghorn) have the derived S781 allele. Sir John’s birth date is not precisely known: we have assumed he was born in 1245 ± 16 CE. He died in 1298.

Extensive genetic and genealogical information for the Stewart family already exists in public-domain databases. The 26 tests listed at YTree.net (https://www.ytree.net/DisplayTree.php?blockID=87, accessed on 30 May 2021) include information about SNP calls and test coverage. Of these tests, 25 were carried out by Family Tree DNA, whose database (https://www.familytreedna.com/public/Stuart, accessed on 30 May 2021), contains additional STR and genealogical data for these individuals. Most of these 25 tests were either BigY-500 tests, with typical coverages of 10 Mbp, or BigY-700 tests, with typical coverages of 15 Mbp. While a more extensive haplotree is available at Family Tree DNA itself (https://www.familytreedna.com/public/y-dna-haplotree/R;name=R-S781, accessed on 30 May 2021), this analysis is restricted to those kits and haplogroups listed at YTree.net for practical purposes relating to ethical permissions and access to relevant data.

#### 3.4.2. Constructing a Data Model

**Figure 4:**
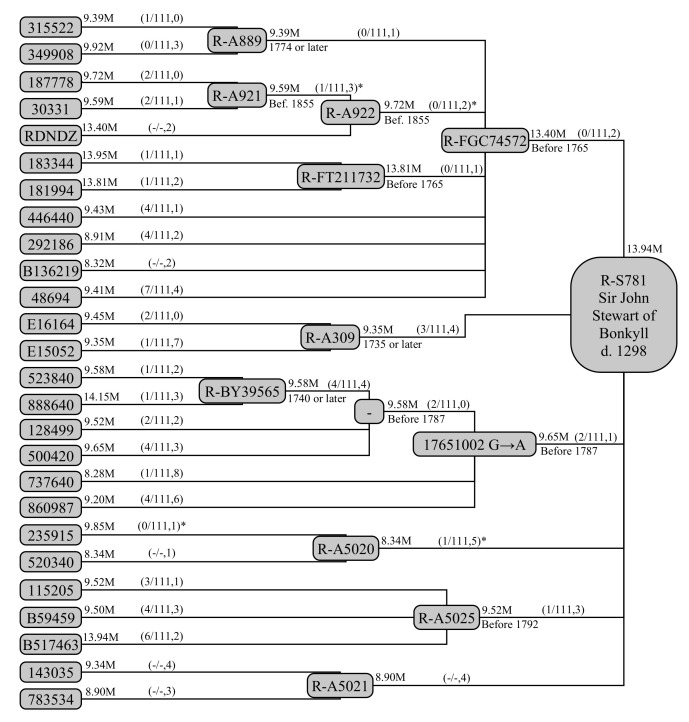
A schematic representation of the example given in Section 3.4. Individual testers and the haplogroups they form are shown as the interconnected grey boxes. Participants are listed using the identifier assigned by their testing company. Measured or estimated coverage, in millions of base pairs, is shown to their upper-right; restrictions known from paper genealogies are shown to their lower-right. Between testers and haplogroups, in brackets, are shown the number of STR mutations that have occurred (*g*), the number of STR markers tested, and the number of SNP mutations that have occurred within the region of testing used for the parent haplogroup ($\bar{b}$). Note that the mutation at GRCh38 position 17651002 is sometimes designated A22007; R-A309 is labelled by Family Tree DNA by the alternative name R-A306. The asterisked genetic distances cannot be uniquely placed in the tree, due to lack of STR results for some testers: these are discarded for this calculation.

These tests and their relationships are shown in Figure 4. The sampling of the Stewart family exemplifies both advantages of this model, and the difficulties in dealing with real-world data.

While a stated coverage exists in the public domain, we do not know exactly which base pairs are covered. The overlap in coverage between tests of the same type is large, so the shared coverage within a haplogroup ($\bar{b}$) can be approximated as being the second-highest coverage among its constituent sub-clades and individual testers. This also necessitates estimation of which SNPs should be counted within this coverage. To estimate this, 2477 BigY tests from Family Tree DNA were examined for coverage. A SNP was counted if it fell within the coverage of 50% of BigY-500 or BigY-700 tests, as appropriate for the haplogroup’s $\bar{b}$. For both the SNP counts and $\bar{b}$ itself, the Y-chromosomal regions PAR1, DYZ19, Yq12 and PAR2 were excised, as was the centromeric region, resulting in a total decrease of ∼940,000 base pairs of coverage for each test. Palindromic regions were retained. We note the defining mutation at GRCh38 position 17651002 belongs on palindromic arm 5, though it is not clear on which end: this mutation is also termed A22007, but we do not use this name in the following, retaining the name used in YTree.net (R-17651002GA) for ease of comparison.

The public genealogical constraints on haplogroups are also limited in this study. While full genealogies are available to the organisers of the Royal Stewarts project, only the most-distant known ancestors (MDKAs) are listed here. Hence, we only know that a relationship has either occurred before a certain date if the two stated MDKAs are different, or that it occurred through an individual or one of their descendants (therefore on or after a certain date) if the two stated MDKAs are the same. We are also relying on the individuals themselves to report the correct genealogy, and some have not included this information.

Several tests are also missing Y-STR results from the public domain. For example, individual RDNDZ has been tested by Full Genomes Corporation (https://www.fullgenomes.com/) and has Y-STR results that are only partly compatible with this analysis. Consequently, this means we cannot securely use STRs to measure the temporal distance between R-FGC74572 and R-A922, nor between R-A922 and R-A921. However, this test is also an example of high coverage (13.40 million base pairs, Mbp) compared to the other R-A922 tests (9.59, 9.72 Mbp; hence $\bar{b}=9.72$ Mbp for R-A922). One SNP from this test has been excised as it is untested in other R-A922 members and would not fall in the combined $\bar{b}$ for R-A922. STR results are similarly missing from R-A5020 and R-A5021.

Beneath R-17651002GA, four testers (523940 and 888640, defining R-BY39565, and 128499 and 500420) share two STR mutations not found in other members of the haplogroup (737640, 860987). These can be used to define an intermediate haplogroup on the basis of these Y-STR mutations, allowing us to identify an intermediate genealogical step and provide its age.

Before continuing, it is worth mentioning what the TMRCA of the R-S781 haplogroup actually means from a computational perspective. While at least two R-S781 sub-clades are related through Sir John himself, the relationship of the other branches to these two individuals are unknown, so could have been at any point before an SNP formed along any of these lines. It is likely that the six branches separated from each other within a few generations (Sir John left five sons with known issue). However, the TMRCA is calculated as the average of each pairwise average of sub-clades (with six branches, there are 15 pairs) and it is possible that the calculated TMRCA is more recent than the global TMRCA by a few decades. This is not encapsulated within the standard errors.

#### 3.4.3. Results

The haplogroups extracted from Figure 4 and shown in Table 5, along with the known constraint from paper genealogies. We begin by calculating ages without the benefit of existing paper-trail information, to check the accuracy of the model. This provides the dates of MRCAs shown in the third column of Table 5. The age of R-S781 is correctly reproduced at 1254 CE, with a 95% confidence interval of 1087–1390 AD or ${}_{-167}^{+136}$ years. This error budget is still dominated by the small number of mutations present in the upper branches of the tree, though fundamental limit set by the uncertainty in the SNP mutation rate is now ∼±67 years, meaning it could quickly become dominant if all tests in the Family Tree DNA database were used. It is worth noting that the testing in this haplogroup is dominated by Family Tree DNA’s BigY-500 tests of ∼9.5 Mbp of useable coverage. As a larger portion of tests become dominated by various longer tests of ∼13.5 Mbp of usable coverage (as is already the case in less-high-profile haplogroups), the uncertainty will be reduced by ∼17% further in a haplogroup of similar size, age and structure.

Applying constraints from genealogical records further down in the tree, we produce the fourth column of Table 5. We can see these constraints change the inferred ages by several decades, most notably R-BY39565 and its upstream haplogroups to R-17651002GA, which become older by 40–60 years. This change also affects the age of R-S781 slightly, making it older by 21 years. This change has minimal effect on the uncertainty budget, reducing it only to ${}_{-165}^{+134}$ years.

Finally, we can apply the constraint R-S781 itself in its MRCA of Sir John Stewart of Bonkyll. We can propagate this down to the immediate sub-clades of R-S781 on a multiplicative basis, since the large number of sub-clades (six) means that the age of R-S781 does not depend too strongly on any one of them. Some correlation will remain, especially with the haplogroups that constrain R-S781 the most (particularly R-FGC74572), therefore these can only approximate the true ages of these haplogroups. These are shown in the final column of Table 5. We can see that this allows much better constraint on small and poorly tested haplogroups, such as R-A5021, where the central estimate has moved by 150 years, and the uncertainty budget shrunk from ${}_{-466}^{+285}$ years to ${}_{-279}^{+201}$ years.

#### 3.4.4. Comparison to Other Methods

A comparison to Y-STR methods for this haplogroup is difficult becaues of its complex structure. For SNPs, a modified version of the approach of [5], which introduces revised weighting and 33 SNP tests, is available at YFull (https://yfull.com/tree/R-S781/, accessed 3 June 2021) and serves as the best available comparison. The additional tests compared to our 26 could not be used here, as the anonymised participant IDs cannot securely be mapped between the two trees, thus it is not possible to unambiguously identify potential duplicate entries.

YFull quotes an age of 598 years for R-S781, with a 95% confidence interval between ∼475 and ∼700 years (${}_{-123}^{+102}$ years). Assuming a zero-point of 1956 AD, this equates to a date of MRCA of 1256 to 1431 AD, providing some tension with the birth date of Sir John of ∼1245 AD. While the quoted uncertainty appears smaller, it must be remembered that the uncertainties do not take into account the full Poisson uncertainty, and the weighting algorithm is not clearly described. Hence, this tension may be resolved by increasing these errors appropriately. Consequently, we cannot easily assess the improved precision of the model over [5], but we can state that there is a considerable improvement in accuracy, at least in this instance.

## 4. Discussion and Conclusions

The examples above demonstrate the use of the revised method (Section 2) in generating accurate and precise TMRCAs from Y-DNA mutation clocks, and demonstrates its improvement on common methods used in the commercial community. Allowing arbitrary combinations of historical data can be included, including genealogical and ancient DNA data, can provide further improvement in the TMRCAs in a self-consistent manner. Steps have been outlined to allow the inclusion of ancient DNA, potentially allowing substantial improvement in the accuracy of TMRCA calculations in more ancient times.

The most significant improvements in the precision of the TMRCAs come from the ability to combine both STR and SNP mutations into a single calculation. The ability to constrain the TMRCAs based on historical data can be important when those ages fall within the overall TMRCA uncertainty budget, as in Example 2. However, the correction for back mutations and multiple mutations in the STR results is crucial in avoiding under-estimated ages, as demonstrated in Example 3.

Even for these small, simple examples, a significant source of uncertainty is the mutation rate applied to the data. With this combined STR/SNP method, the mutation rate uncertainty dominates the error budget for most real-world situations involving larger and more-complex examples than those presented here. This acts as a theoretical limit to the precision of TMRCA calculations. If these mutation rates can be improved, the advantages of this combined model over either STR-only or SNP-only models will become even more significant.

While every effort has been made to be comprehensive, this work does not deal with many practical aspects of adapting this method to a real Y-DNA haplotree. As a closing comment, I would advise users of this method to approach their data with an abundance of caution: the small numbers of mutations involved in many of these calculations mean a single mutation can make a significant difference to the TMRCAs involved, thus whether mutations are counted correctly is as important a factor in obtaining accurate TMRCAs as whether the correct method is used.

## Figures and Tables

**Table 1:** Computed probabilities P(g|m) of obtaining genetic distance *g* from *m* mutations.

*g*	*m* = 0	*m* = 1	*m* = 2	*m* = 3	*m* = 4	*m* = 5	*m* = 6	*m* = 7	*m* = 8	*m* = 9	*m* = 10
0	1.000	0.000	0.479	0.024	0.364	0.003	0.302	0.006	0.261	0.009	0.230
1	0.000	0.962	0.032	0.725	0.020	0.607	0.009	0.526	0.014	0.465	0.020
2	0.000	0.032	0.483	0.005	0.485	0.017	0.454	0.012	0.418	0.017	0.384
3	0.000	0.004	0.004	0.242	0.006	0.303	0.015	0.316	0.013	0.311	0.018
4	0.000	0.001	0.001	0.003	0.122	0.006	0.182	0.013	0.210	0.013	0.221
5	0.000	0.000	0.000	0.001	0.003	0.061	0.005	0.106	0.011	0.135	0.012
6	0.000	0.000	0.000	0.000	0.000	0.002	0.031	0.005	0.061	0.008	0.084
7	0.000	0.000	0.000	0.000	0.000	0.000	0.001	0.016	0.003	0.034	0.007
8	0.000	0.000	0.000	0.000	0.000	0.000	0.000	0.001	0.008	0.003	0.019
9	0.000	0.000	0.000	0.000	0.000	0.000	0.000	0.000	0.001	0.004	0.002
10	0.000	0.000	0.000	0.000	0.000	0.000	0.000	0.000	0.000	0.000	0.002

**Table 2:** Results for age of E. in Example 1, in the scenario where the age of D. is known.

Method	Resulting TMRCA (years, 95% c.i.)	
Input	300	
This work	390	(238–614)
SNPs only [5]	610	(293–927)
SNPs only [5] with Poisson correction	581	(318–961)
STRs only [1]	298	(123–595)

**Table 3:** Results for age of “Emn” in Example 2.

Method	Resulting TMRCA (years, 95% c.i.)	
Input	1100	
This work, excluding paper genealogy	1071	(798–1413)
This work, including paper genealogy	1221	(948–1563)
SNPs only [5]	1220	(837–1603)
SNPs only [5] with Poisson correction	1190	(865–1630)
STRs only [1]	980	(700–1365)

**Table 4:** Results for the MRCA in Example 3.

Method	Resulting TMRCA (Years, 95% c.i.)	
Input	4000	
This work	3667	(3060–4375)
This work, SNPs only final step	3873	(3090–4822)
SNPs only [5]	4084	(2757–5411)
This work, STRs only final step	3419	(2549–4535)
STRs only [1] *	2560	(2000–2695)

* Uncertainty range is an under-estimate.

**Table 5:** Date of MRCA estimates in Example 4.

Haplogroup	Known MRCA	Estimated MRCA (Years CE) *					
		No Paper		Constraint Up		Constraint Down	
R-A889	≥1774	1825	(1666–1911)	1842	(1778–1914)		
R-A921	<1855	1798	(1628–1896)	1785	(1620–1851)		
R-A922	<1855	1491	(1101–1725)	1476	(1089–1703)		
R-FT211732	<1765	1491	(1101–1725)	1476	(1089–1703)		
R-FGC74572	<1765	1592	(1473–1687)	1601	(1484–1693)	1636	(1533–1716)
R-A309	≥1735	1625	(1388–1782)	1612	(1382–1726)		
R-BY39565	≥1740	1742	(1548–1861)	1684	(1513–1738)		
${}^{†}$	<1787	1532	(1335–1679)	1477	(1290–1608)		
R-17651002	<1787	1443	(1252–1591)	1393	(1208–1531)	1454	(1307–1569)
R-A5020		1764	(1434–1912)	1764	(1434–1912)	1819	(1585–1924)
R-A5025	<1792	1602	(1410–1741)	1602	(1410–1740)	1642	(1473–1760)
R-A5021		1441	(975–1726)	1441	(975–1726)	1590	(1311–1791)
R-S781	∼1245	1254	(1087–1390)	1233	(1068–1367)	1245	(1229–1261)

* The first column includes no paper-trail constraint. The second column includes paper-trail constraints, not including the fixed age of R-S781 itself. The third column recomputes the age of sub-clades from the fixed age of R-S781, as described in the text. ^†^ This is the unnamed haplogroup formed from STR results.

## Data Availability

The code used to generate the examples in this paper is available at https://github.com/iain-mcdonald/TMRCA.

## Appendix A. A Priori Calibration

This Appendix discusses the main parameters requiring calibration before computation, and their validity as constants.

### Appendix A.1. Defining “Present Day”

By linking each phylogenic node to the birth date of the tester or MRCA, we simplify historic referencing, but this is also advantageous because genetic chimerism on a scale large enough to change in a Y-DNA test is likely to result from a mutation occurring in utero, rather than in vivo. This is in contrast to actual inheritance, where in vivo germ-line mutations accumulate on the Y chromosome and are passed on to offspring [23].

Commercial genetic testers tend to be more elderly than the average population. A poll of 131 testers revealed a mean age of 64 years and 95% confidence interval of 35–91 years. This confidence interval should therefore suffice for any modern testers where birth dates remain unknown, and should be added on to any TMRCA computed from the present day.

### Appendix A.2. Allowing Back Mutations and Recurrent Mutations of Y-Snps

Convergent mutations found in Y-STRs (Section 2.5.3) also occur rarely in Y-SNPs. Given a point mutation rate of ∼8 × 10${}^{-10}$ SNPs per base pair per year (Appendix A.4), and the billions of men alive today, every SNP can be considered recurrent at some level. However, it is clear that the mutation rate is only valid when averaged over large regions of the Y chromosome, and that individual base pairs can have a higher tendency to mutate. Mutations are ∼9–12.3 times more common in CpG sites, and ∼16 times higher in the heterochromatic regions around the centromere [24,25]. Such sites may include gene conversion hotspots: “clear evidence of GC-biased gene conversion in the palindromes and a tendency for gene conversion to revert mutations to their ancestral state” has been found [26].

The principle of TMRCA calculations requires matching the conditions under which SNPs are counted to the conditions used to derive a mutation rate. As highly recurrent base pairs are excised from mutation-rate estimations, they should also be self-consistently removed from TMRCA calculations and excised from the subset of base pairs $\bar{b}$. An approximate rule of thumb might be that SNPs that recur or back mutation once per few hundred tests should be considered unstable, but this varies depending on the age of the haplogroup and source of mutation rate used.

### Appendix A.3. Identifying Complex Variants and Read Errors

Errors in reading or mapping the DNA sequence can also masquerade as recurrent or frequently back-mutating SNPs. The short read length and imperfect mapping quality of most sequencing tests can also lead to more complex mutations masquerading as SNPs, or a series of SNPs due to bad mapping, even when the nominal mapping quality of the read is high. This effect is strongest in highly repetitive sections of the Y chromosome, such as the centromere and DYZ19, and it may ultimately be best to mask these problematic regions from the analysis (few mutation rates have been defined for these regions, in any case). This may also affect treatment of the palindromic arms, depending on how variants in these regions are called.

Typically, mapping errors result in sequences of several SNPs clustered in a small region, comparable to the read length (typically ∼100 base pairs in commercial sequencing tests). To identify how likely it is to obtain two SNPs within a specific position difference, a Monte Carlo simulation was set up using a real list of callable loci from a commercial sequencing test. Twelve SNPs were inserted into this set of loci at random and the smallest position difference between them was calculated. This was repeated 100,000 times. In most tests, the closest mutations were still separated by millions of base pairs. In 1% of cases, the mutations were closer than ∼40,000 base pairs, and in 0.1% of cases they were closer than ∼150 base pairs; for 50 SNPs, these mimimum distances decrease to ∼19,000 and ∼30 base pairs. Consequently, two or more SNPs found within the same 100 base-pair region can be identified as forms of multi-nucleotide polymorphisms or other complex mutations with high confidence, and should be removed from the TMRCA analysis. For basal haplogroups (e.g., haplogroup A00), it is possible that a small number of genuine SNPs may occur within ∼100 base pairs of each other, but these would be exceptional circumstances.

Complex variants like these have been identified in the literature (e.g., [21], their Supplementary Table 10), although a physical mechanism for their generation has not been clearly identified. Published rates for non-recurrent multi-nucleotide polymorphisms (MNPs) are 0.9% of the SNP rate [25], and 1.3% when other complex mutations are included [26]. The generalised rate of insertions and deletions (indels) is 11.5% of the SNP rate [25], or 10.5% and 11.2% when reported separately in the ampliconic and X-degenerate regions [26].

### Appendix A.4. Defining a SNP Mutation Rate

Several estimates for the SNP point mutation rate exist in the literature, averaging around 8 × 10${}^{-10}$ SNPs bp${}^{-1}$ yr${}^{-1}$, thought to be fairly constant. However, low-level variability may become important when creating precise TMRCAs. These include differences in mutation rate in different regions of the chromosome; carcinogenic mutations caused by environment and diet; changes to the average number of mitoses or meioses per year, primarily due to changing generation lengths; and evolutionary deleterious mutations that could cause a difference between the short-term mutation rate and the long-term substitution rate. Consequently, care must be taken to ensure the mutation rate is appropriate to the coverage of the test and the prehistoric age and population in question.

By comparing mutation rates derived from different populations, we can determine how (or even if) environment, diet, generation length and haplogroup affect the mutation rate. By comparing mutation rates derived from modern populations and from ancient DNA, we can determine the effect of mutation versus substitution rates.

#### Appendix A.4.1. Effect of Rapid Population Growth on Effective Mutation Rate

First, let us explore a statistical effect, whereby larger sub-clades tend to have more mutations on average than their smaller counterparts. To exemplify why this occurs, one can imagine a male population which doubles each generation, each father having two sons, with one mutation forming on average every four generations. In the first generation, one son sporadically gains a SNP mutation, and his descendants go on to form a sub-clade, *A*, containing half of the parent clade. Meanwhile, another clade-defining mutation, *B*, occurs in a fifth-generation great-great-grandson, and forms 1/32 of the parent clade. In those extra four generations, roughly half of *A* will have gained a second, additional mutation compared to *B*.

This becomes important in populations where the timescale for population growth exceeds the timescale for mutation of the specified test ($1/b\mu_{\mathrm{SNP}}$). To demonstrate this, we have modelled populations with different growth rates (λ), adopting a Poisson distribution for the number of reproducing sons from each lineage, and assuming $1/b\mu_{\mathrm{SNP}}=4$ generations per SNP. Each test was run for 16 generations (to represent a typical cultural timescale of 500–600 years). For each λ between 1.0 and 2.4, in steps of 0.01, populations were modelled until ∼100 families with multiple extant sub-clades were generated, and the difference between the average number of SNPs in the largest and smallest clades was recorded for each.

For populations with λ≲1.25, no significance difference in the average number of SNPs was recorded. Very few tests showed larger clades having smaller numbers of SNPs, whereas a small tail of tests showed larger clades having more SNPs. As λ was increased above 1.25, there was a rapid growth in the excess number of SNPs in larger clades. This excess asymptotes to around 2.3 extra SNPs in larger clades, for λ≳1.8, with a standard deviation of around 1.0–1.5 SNPs.

Thus, a difference in the average number of SNPs between two brother haplogroups need not necessarily imply a difference in the overall mutation rate between them. However, this can lead to issues with causality, and appropriate measures may be needed to address this factor (Section 2.3).

#### Appendix A.4.2. Checking for Variation in the Modern Mutation Rate

In this comparison, we do not take into consideration mutation rates scaled from autosomal pedigree studies. Such rates are subject to the additional and often poorly quantified uncertainty of accounting for male-specific processes. For example, [27] identify a mutation rate of $6.17\times 10^{-10}$ SNP bp${}^{-1}$ yr${}^{-1}$ (95% CI: 4.39–7.07 $\times 10^{-10}$), significantly lower than the studies cited below.

One of the first measures of the SNP mutation rate [28] used 10.15 Mbp tests of two Chinese individuals, separated by 13 generations. A mutation rate was found of $1.0\times 10^{-9}$ SNP bp${}^{-1}$ yr${}^{-1}$ was found, but with a large confidence interval (95% CI: $3.0\times 10^{-10}$–$2.5\times 10^{-9}$).

The first application of pedigree studies to commercial Y-DNA tests [5], used 41 samples from 14 genealogies, separated by up to 23 generations averaging 32.1 yr gen${}^{-1}$. They identify a mutation rate of $7.98\times 10^{-10}$ SNP bp${}^{-1}$ yr${}^{-1}$, with a much smaller confidence interval (95% CI: 6.32–9.84 $\times 10^{-10}$).

Sequencing data of 482 Icelandic family branches covering 47,123 years of genealogies (34.5 years gen${}^{-1}$) and sampling the majority of the male-specific Y chromosome (MSY; 21.3 Mbp) were analysed to yield a combined rate of $8.33\times 10^{-10}$ SNP bp${}^{-1}$ yr${}^{-1}$ (95% CI: 7.57–9.17 $\times 10^{-10}$) [21]. A faster rate was found for the combination of the X-transposed, X-degenerate and ampliconic sections of the Y chromosome (15.2 Mbp; $8.71\times 10^{-10}$ SNP bp${}^{-1}$ yr${}^{-1}$; 95% CI: 8.03–9.43 $\times 10^{-10}$), with some evidence of a slightly lower rate in the palindromic regions (6.1 Mbp; $7.37\times 10^{-10}$ SNP bp${}^{-1}$ yr${}^{-1}$; 95% CI: 6.41–8.48 $\times 10^{-10}$; *P* = 0.04), hypothesised to be due to gene conversion between the paralogous sequences of the palindromic arms, and which could be related to variations in the amplicon copy number [29]. Crucially, [21] did not identify any significant differences between the X-transposed region (which is not covered by most other studies) and the X-degenerate or ampliconic regions, indicating that the mutation rate is constant when sufficiently large regions of the MSY are considered. They also did not identify any significant differences in mutation between haplogroups, with the exception of a possible statistical anomaly in haplogroup E1b1. The average family branch length was only 98 years, meaning there should be extremely little influence from evolutionary selection.

#### Appendix A.4.3. Checking for Variation in the Mutation Rate with Time

A (now commonly used) masked region of 9.99 Mbp was applied by [30] to sequencing of 69 males from nine populations. By assuming the Q-L54 haplogroup represents the last common ancestor of the first settlers of the Americas (15 kya), they established a mutation rate of $8.2\times 10^{-10}$ SNP bp${}^{-1}$ yr${}^{-1}$ (95% CI: 7.2–9.2 $\times 10^{-10}$) over this period. The inclusion of carbon-dated remains (Anzick-1 [31]) provided a more accurate mutation rate for the Q-L54 sub-clade Q-Z780 of $8.31\times 10^{-10}$ SNP bp${}^{-1}$ yr${}^{-1}$ (95% CI: 6.6–10.4 $\times 10^{-10}$) [5]. It is noteworthy that this already reproduces the mutation rates found in pedigree studies, even those of very different haplogroups [21], hence it can be noted that the Y-SNP molecular clock remains accurate even over paleolithic timescales.

Similarly, by assuming I-M26 represents the last common ancestor of arrivals to Sardinia (7.7 kya), a mutation rate of $5.3\times 10^{-10}$ SNP bp${}^{-1}$ yr${}^{-1}$ has been established in a low-coverage dataset [32]. This rate increased to $6.5\times 10^{-10}$ SNP bp${}^{-1}$ yr${}^{-1}$ when the coverage was increased to 8.97 Mbp of the X-degenerate regions. While no uncertainties are given, this is clearly in tension with the Poznik et al. result, but this may be due to the approximate calibration of the relevant archaeological date.

The “missing” mutations from sequencing an archaeological Siberian individual (Ust’-Ishim; 45 kya, and ancestral to haplogroup K(xLT)) have provided a mutation rate of $7.6\times 10^{-10}$ SNP bp${}^{-1}$ yr${}^{-1}$ (95% CI: 6.7–8.6 $\times 10^{-10}$) [33]. While this is a lower rate than most pedigree studies, it still agrees within the stated confidence intervals. A similar study [34], using results from two ancient DNA samples from haplogroups Q1 and Q2b, identify a mutation rate of $7.4\times 10^{-10}$ SNP bp${}^{-1}$ yr${}^{-1}$ (95% CI: 6.3–9.5 $\times 10^{-10}$) over ∼12 kyr of history.

The same Ust’-Ishim remains, plus ancient DNA from Loschbour [35], were used to estimate a global mutation rate of $7.16\times 10^{-10}$ SNP bp${}^{-1}$ yr${}^{-1}$ (95% CI: 6.19–8.15 $\times 10^{-10}$) [36]. Applying this rate to modern individuals, the overall mutation rate was not found to vary with statistical significance between haplogroups (including the basal A00 clade), showing that Y-SNP mutation must be indistinguishable from evolutionary neutrality. By contrast, the variation in total branch length of haplotree suggests mild (perhaps ±10–20%) variation in the mutation rate between haplogroups is possible, with the suggested cause being replication timing during cell division caused by differing amounts of heterochromatin [37].

The stability of the Y-DNA point mutation rate can also be investigated using phylogenic studies of the great apes. While full sequencing of the great-ape Y-chromosomes has only recently been completed, the relative length of branches defining each species (including humans) is roughly the same [38], indicating only small changes in the evolutionary rate of Y-SNP formation.

In summary, despite the limited data available from truly ancient DNA samples, the evolutionary substitution rate of Y-SNPs appears functionally identical to the pedigree-based mutation rate of Y-SNPs, both being ∼8 $\times 10^{-10}$ SNPs bp${}^{-1}$ yr${}^{-1}$. Several conditions are linked to mutations on the Y chromosome, but these are mostly related to male fertility and mostly occur through large-scale gene deletion, e.g., [39]. Consequently, it seems reasonable that SNP mutations on extant (i.e., reproducing) male lines can be treated as evolutionarily neutral, with a mutation rate constant across time and space to within the present uncertainties of its measurement. However, while the Y chromosome can be stable to even large deletions (e.g., 118 kbp; [40]), that same evolutionary neutrality is not yet robustly evidenced for MNPs, insertions, deletions, complex mutations and Y-STRs.

### Appendix A.5. Defining a Set of STR Mutation Rates

#### Appendix A.5.1. Defining a Generation Length

For historical reasons, the mutation rate for Y-STRs tends to be described in mutations per generation. However, Y-STR mutation frequency correlates with paternal age [12]. Thus, we need the generation length (the average age of a father at each child’s birth) to derive calendar TMRCA dates.

A useful summary is provided by [23], whose Figure 4 shows the variation in mean paternal age at conception over time in Icelandic genealogies. A stable figure of ∼35 ± 1 yr gen${}^{-1}$ exists before circa 1900, after which advances in healthcare and contraception during the 20th Century decrease the mean to 28 yr gen${}^{-1}$ during the 1970s, after which rates rise again. Given most commercial testers’ births predate the worst of these variations (Appendix A.1), the historic rate should generally suffice. Given the pre-Industrial Icelandic population was a largely agrarian society, it may be reasonable to extend this estimate across all populations since the Neolithic. Slightly lower rates, of order 31–35 yr gen${}^{-1}$, have been found by other studies (e.g., [41,42]), but these rates seem to have rough consistency across all cultural populations.

For this historical analysis, we therefore recommend a mean of around 33 years (95% CI: 29–37 years) which is roughly constant throughout time. On top of this, for small numbers of generations, there will be a random scatter introduced at a generational level, for which we can assume a Gaussian width of ∼8 years in each generation [43], hence 8$\sqrt{N}$ years in *N* generations.

#### Appendix A.5.2. Literature Estimates of Y-STR Mutation Rates

Mutation rates for individual Y-STRs vary by orders of magnitude, meaning a blanket mutation rate for all Y-STRs will lead to an inaccurate estimate of the overall TMRCA, especially where the number of Y-STRs is variable, or only a few Y-STRs have mutated. Consequently, literature estimates of mutation rates of individual markers are often discrepant between studies and many have large associated uncertainties.

The mutation rates of individual Y-STRs correlate most strongly with their length: Y-STRs with longer repeat units will mutate faster, as will Y-STRs with more repeats (e.g., [14,44]; note that the mutation rate refers to the whole Y-STR, not the rate per base pair). This correlation with length extends to per-allele rates of specific Y-STR markers, leading to small differences in the mutation rate between haplogroups of ∼20% [44]. While these can be used to predict per-locus Y-STR mutation rates (e.g., [45]), it is preferable to obtain mutation rates from homogeneous studies of many Y-STR markers (cf., [46]). Mutation rates for 186 Y-STRs among 2000 father-son pairs, with 95% CIs of ±20% [8] and a very similar overall rate to [45]. Data from the 1000 Genomes Project includes 702 polymorphic Y-STRs out of a total of 4500 Y-STRs: the mutation rates of these Y-STRs were pinned to the Y-SNP tree by [47] using a Y-SNP mutation rate of 3 × 10${}^{-8}$ per generation from [21]. Over 81 Y-STR markers common to both studies and to several commercial testing companies, the derived per-locus mutation rate is 35% slower than [45].

Section 2.5.3 discussed biased directionality in Y-STRs, where $w_{+}\neq w_{-}$. If this occurs, either through preferential structural stability within the DNA molecule, or the non-neutral evolutionary selection of some Y-STR alleles, then genetic drift will occur back towards the most stable/fittest locus, invalidating variance-based mutation rates and TMRCAs (e.g., [2,48]). With the tree-based approach outlined in Section 2.5, this problem is largely circumventable. However, the method still fails for trees with large gaps, e.g., [49], and non-random or non-neutral mutation may explain the differences in the pedigree-based mutation rates of [8], and the evolutionary rates of [47] (see review, [50]). Consequently, it is likely that a maximum time limit should be associated with inclusion of Y-STRs in the TMRCA calculation, and either an exponential correction function on P(t|STR), a revised mutation rate (μ) at large *t*, or (and this is mathematically preferable) a Gaussian tapering to P(g|m) that limits large *g* at large *m*.
